# Effects of Capsaicin on Migration and Alkaline Phosphatase Activity of Dental Pulp Cells

**DOI:** 10.1055/s-0044-1782191

**Published:** 2024-05-02

**Authors:** Kittipot Khonglim, Boontharika Chuenjitkuntaworn, Yukihiko Tamura, Pornpoj Fuangtharnthip

**Affiliations:** 1 Department of Advanced General Dentistry, Faculty of Dentistry, Mahidol University, Ratchathewi, Bangkok, Thailand; 2 Department of Cariology and Operative Dentistry, Division of Oral Health Sciences, Graduate School of Medical and Dental Sciences, Tokyo Medical and Dental University, Tokyo, Japan

**Keywords:** capsaicin, dental pulp, cell migration, alkaline phosphatase activity

## Abstract

**Objectives** Dental pulp, a specialized mesenchymal tissue within teeth, is pivotal in dental health and tissue repair. Capsaicin, the primary pungent component of chili peppers, is known for its diverse pharmacological properties. While capsaicin's effects on various cell types have been studied, its impact on dental pulp cells remains relatively unexplored. This study investigated the influence of pure capsaicin extract on dental pulp cell behavior, focusing on cell viability, proliferation, migration, and alkaline phosphatase (ALP) activity.

**Materials and Methods** Capsaicin solution was prepared and diluted to various concentrations (1 nM, 0.01 µM, 0.1 µM, 1 µM, 10 µM, and 100 µM), then was tested on rat dental pulp cells (RPC-C2A). Cell viability and proliferation were assessed using the MTT assay. Boyden chamber tests and wound healing were used for evaluating cell migration. The activity of ALP was determined to show cell function during dental pulp repair.

**Statistical Analysis** The data were analyzed using a one-way analysis of variance or an independent-sample Kruskal–Wallis, followed by multiple comparison tests.

**Results** Capsaicin of 100 µM exhibited cytotoxicity, whereas those with lower concentrations stimulated cell proliferation. Wound healing assays revealed increased cell migration, particularly when cultured with 1 nM capsaicin ( *p* = 0.002). Boyden chamber assays demonstrated enhanced cell invasion without statistical significance. ALP activity of dental pulp cells increased significantly at 1 nM ( *p* < 0.001) and 1 µM ( *p* = 0.021) capsaicin concentrations, indicating potential dentinogenesis and pulp repair.

**Conclusion** Capsaicin of lower concentrations, less than 10 µM, is likely to promote proliferation, migration, and ALP activity of dental pulp cells. Our findings offer potential applications for capsaicin as a medication for dental pulp repair.

## Introduction

Dental pulp, a highly specialized mesenchymal tissue within the tooth, plays a crucial role in maintaining dental health and facilitating the repair and regeneration of dental tissues. It contains odontoblasts, which are specialized cells involved in dentinogenesis. 1 2 3 Dental pulp injuries due to carious infection and dental trauma are very common. Dental scientists have been always searching for the way to preserve pulp vitality and promote its healing. 4 It is widely known that dental pulp stem cells, when properly induced, can differentiate into other cells including adipocytes, chondrocytes, and osteoblasts, and neurons. 5 6 7 Therefore, studies on dental pulp cells could be beneficial to dental and medical regenerative therapy.

Capsaicin, known chemically as 8-methyl-N-vanillyl-6-nonenamide, is the primary pungent component of chili peppers. Over the years, capsaicin has attracted significant research interest due to its diverse pharmacological and toxicological properties. It has been extensively studied for its effects on various cell types, revealing its potential in regulating cell proliferation, migration, differentiation, and apoptosis. 8 9 Furthermore, capsaicin has demonstrated antibacterial and antivirulence activities against pathogens such as *Streptococcus pyogenes* . 10

One of the notable applications of capsaicin is its use as a topical analgesic for chronic pain relief, owing to its antioxidant and anti-inflammatory effects. 11 12 13 Activation of the transient receptor potential vanilloid subtype 1 (TRPV1) by capsaicin on C-fibers and A-delta fibers contributes to its analgesic actions. 14 Recent research has shown that TRPV1, which can be activated by capsaicin, is expressed not only in sensory neurons but also in various non-neuronal cells such as osteoblasts and osteoclasts, including dental pulp cells. 15 TRPV1 is known to regulate osteoblast and osteoclast differentiation and function, and its blockade has been shown to protect against bone loss. 16 These suggest that TRPV1 activation by capsaicin may play a role in regulating cell differentiation pathways in dental pulp cells.

While the effects of capsaicin on various cell types have been explored, its specific impact on dental pulp cells remains relatively understudied. Previous studies utilizing capsaicin gel formulations have demonstrated stimulatory effects on dental pulp cell proliferation at low doses but toxicity at higher doses. 17 However, the specific effects of pure capsaicin extract on dental pulp cells have not been fully elucidated. Therefore, this study aimed to investigate the effects of pure capsaicin extract on the proliferation, migration, and alkaline phosphatase (ALP) activity of dental pulp cells.

## Materials and Methods

### Preparation of Capsaicin Solution

A total of 0.0152 g capsaicin (Sigma, St. Louis, Missouri, United States) was dissolved with a volume of 50% dimethyl sulfoxide in 10 mL culture medium for 24 hours and filtered by 0.47-µm syringe filter to obtain 10 mM capsaicin solution. Culture medium was prepared with Dulbecco's modified Eagle's medium (DMEM; Gibco, Carlsbad, California, United States), supplemented with 10% fetal bovine serum, sodium bicarbonate (NaHCO _3_ ), and penicillin at 10,000 unit/mL. The 10 mM capsaicin solution was diluted with DMEM to make different concentrations of 1 nM, 0.01 µM, 0.1 µM, 1 µM, 10 µM, and 100 µM capsaicin (w/v) before use.

### Rat Dental Pulp Cell Culture and Treatment

RPC-C2A cells, a clonal cell line derived from the dental pulp of rat incisor, were kindly gifted by Professor Shohei Kasugai, Tokyo Medical and Dental University, Japan. RPC-C2A cells were cultured in DMEM and incubated at 37°C in a humidified atmosphere of 95% air and 5% CO _2_ . The culture medium was changed every 2 days until 80% confluence. The cells were subcultured to be used for the studies. The treatment groups were cultured with different concentrations of capsaicin and compared with a control group that did not receive capsaicin treatment but cell medium only.

### Cell Viability and Proliferation: MTT Assay

Cell proliferation test was assessed by using a 3-(4,5-dimethylthiazol-2-yl)-2,5-diphenyltetrazolium bromide (MTT) assay (Sigma, St. Louis, Missouri, United States). The RPC-C2A cells were seeded in 96-well plates at a density of 2 × 10 ^3^ cells per well in 100 µL of culture medium. After the cells were recuperated overnight, the cells were treated with 100 µL of capsaicin solutions with different concentrations of 1 nM, 0.01 µM, 0.1 µM, 1 µM, 10 µM, or 100 µM. On days 1, 3, 5, and 7, the RPC-C2A cells were tested with MTT assay by adding 50 µL of MTT solution into each well and incubating for 2 hours after rinsing with 100 µL of phosphate buffer solution (PBS). Then, 100 µL of isopropanol was added into each well and placed on a shaker for 30 minutes. The absorbance was measured at 570 nm by a microplate reader.

### Wound Healing Assay

Cell migration was observed under the wound healing model on monolayer cells. The RPC-C2A cells were seeded on 24-well plates at a density of 5 × 10 ^4^ cells in 500 µL medium per well and incubated 24 hours overnight at 37°C and 5% CO _2_ , allowing cells to adhere and spread on the culture plate. A scratch wound was created in a straight line using a P-1000 pipette tip at the center of the well. The scratch wound area was observed under an inverted microscope, and photographs were taken at the beginning of time (T0). The width of the wound area was measured with ImageJ and accepted when it was 1.4 ± 0.1 mm wide.

The cell cultures with a scratch wound were treated with 1 nM, 0.01 µM, 0.1 µM, 1 µM, or 10 µM capsaicin solutions (nontoxic concentrations from the MTT assay). The control group was treated the same manner with DMEM medium without capsaicin. At 8 (T8) and 16 (T16) hours, the cells were fixed with methanol solution, stained with 10% Giemsa stain for 10 minutes at room temperature, and observed under an inverted microscope. Three migrated areas from the plate of each group were measured and statistically analyzed.

### Migration Assay: Boyden Chamber Assay

In migration assay, a 6.5-mm transwell (Corning Transwell, New York, United States) with 8.0-µm pore polycarbonate membrane insert, TC-treated, was used in this study. The RPC-C2A cells were seeded in 24-well plates at a density of 5 × 10 ^3^ cells per well in 100 µL of culture medium on top of the filter membrane in a transwell insert and incubated for 10 minutes at 37°C and 5% CO _2_ to allow the cells to settle down. A 600 µL of 1 nM, 0.01 µM, or 0.1 µM capsaicin solution was added to the bottom of the lower chamber without moving the transwell insert and avoiding bubbles.

After 24-hour incubation, the culture medium was removed, and 600 µL of 70% ethanol was put into the well for 10 minutes to allow cell fixation. The transwell insert was removed from the 24-well plate, allowed to dry for 10 minutes, and placed back in a new 24-well plate to stain with 600 µL of 10% Giemsa stain for 10 minutes at room temperature. The stained membranes were observed and photographed under an inverted microscope. The number of cells that had migrated through the membrane and attached to the underside were counted in the area of 2.0 × 2.5 mm using the ImageJ program.

### Determination of Alkaline Phosphatase Activity

RPC-C2A cells were seeded at 5 × 10 ^3^ cells/mL in 24-well plates. After overnight incubation, the medium was replaced by the serum-free medium at 3-hour intervals twice to wash out the serum. Cells were treated with 1 nM, 0.01 µM, 0.1 µM, or 1µM capsaicin solution for 5 days. Then, the cells were rinsed twice with PBS, scraped in 200 µL of alkaline lysis buffer (10 mM tris–HCL, 2 mM MgCl _2_ , 0.1% Triton-X-100, pH 10), and kept in −20°C condition until use. The samples were incubated in the buffer containing 2 mg/mL *p* -nitrophenyl phosphate in 0.1 M 2-amino-2-methy-1-propanol, 2 mM MgCl _2_ , pH 10.5 for 30 minutes at 37 °C. The enzyme reaction was stopped by adding 0.8 mL 50 mM NaOH to each well, and absorbance was measured at 405-nm wavelength. The experiment was repeated three times.

## Data Analysis

Statistical analyses were performed using SPSS software (version 18.0, Standard Software Package Inc., United States). The MTT assay, Boyden chamber assay, and ALP activity data were analyzed using a one-way analysis of variance, then applied with the Tukey's Honest Significant Difference (HSD) and Dunnett T3 test, compared with the control group. Due to failure in the normality test, the wound healing assay was analyzed using an independent-sample Kruskal–Wallis test, followed by the Mann–Whitney test compared with the control group.

## Results

### Cell Viability and Proliferation: MTT Assay

**Fig. 1:**
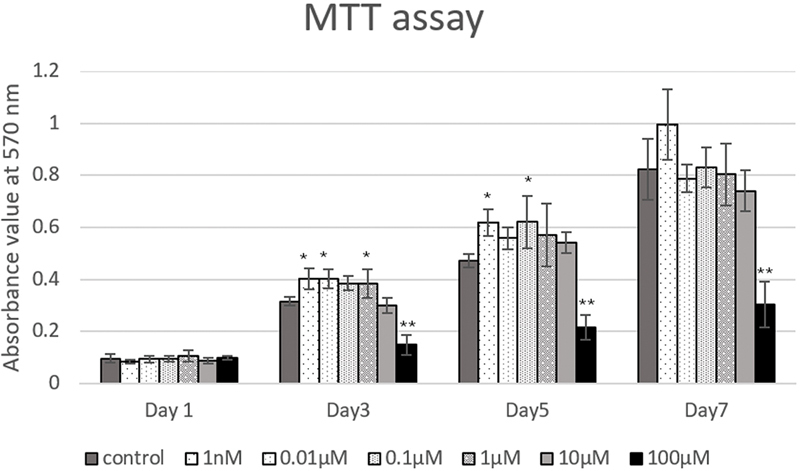
Cell viability of RPC-C2A dental pulp cells treated with capsaicin using MTT assay. No significant difference is found on day 1. On days 3, 5, and 7, the 100 µM group shows a significantly decreased absorbance value. The 1 nM, 0.01 µM, and 1 µM capsaicin treatments have increased cell viability on day 3. The 1 nM and 0.1 µM capsaicin groups show a significant increase in cell viability on day 5. No significant difference is found on day 7 (except 100 µM). Data are representatives of three independent experiments and are shown as the mean ± standard deviation of four to six samples. Asterisks *, ** show significant differences compared with the control group at *p* < 0.05, *p* < 0.01, respectively.

The data of the MTT assay are shown in Fig. 1 . On day 1, all capsaicin-treated groups showed no significant difference in cell viability compared with the control group. However, the 100 µM group exhibited a significant decrease in cell viability from day 3 to day 7 when compared with the control group. Interestingly, on day 3, the 1 nM, 0.01 µM, and 1 µM groups demonstrated a significant increase in cell viability. On day 5, increased cell viability was observed in the 1 nM and 0.1 µM capsaicin groups. On day 7, no significant differences were observed in all the capsaicin-treated groups, except for the decrease observed in the 100 µM group, when compared with the control group.

### Wound Healing Assay

**Fig. 2:**
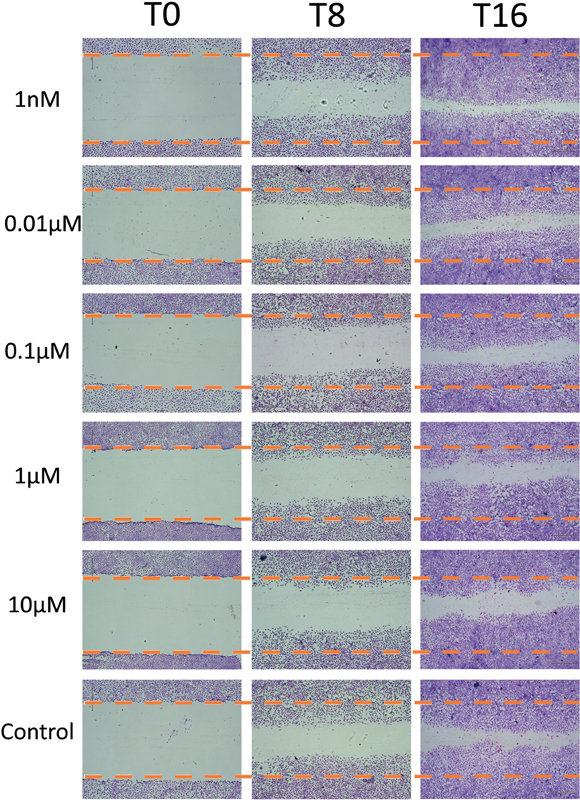
Micrographs of RPC-C2A cells treated with capsaicin concentrations (1 nM–10 µM). Observation under inverted light microscopy was done at 0 (T0), 8 (T8), and 16 hours (T16) after the wounds were made by scratching the cell culture with a pipette tip. Wound closure with migrating cells was found during the observation time. Dotted lines indicate the beginning scratch line at T0.

Cell migration was evaluated in the wound healing assay at 8 and 16 hours after scratching the cell monolayer. Micrographs were taken at three time points: start 0, 8, and 16 hours of the experiment. Fig. 2 displays the micrographs of each group, showing the migration of cells toward the scratched areas in a timely manner. By the 16-hour time, the wounds were almost closed due to cell migration.

To quantify cell migration and wound closure, the micrographs were analyzed using ImageJ. At 0 hour, the average size of the scratch wound area was 5.399 ± 0.597 mm ^2^ . The measurements of migrating areas are presented in Table 1 . At 8 hours, there was no statistically significant difference observed among all groups ( *p* = 0.483), indicating similar levels of migration. However, at 16 hours, the group treated with 1 nM capsaicin exhibited a significant increase in migrating area (4.628 ± 0.418 mm ^2^ ) compared with the untreated cell group ( *p* = 0.002). On the other hand, the other capsaicin groups (0.01, 0.1, 1, and 10 µM) did not show any significant difference compared with the untreated group in terms of migration and wound closure.

**Table 1:** Measurements of cell migrating area at 8 (T8) and 16 hours (T16)

	8 h (T8)		16 h (T16)	
	Migrating area (mm ^2^ ) Mean ± SD	*p* -Value	Migrating area (mm ^2^ ) Mean ± SD	*p* -Value
Control	2.244 ± 0.760	0.483	3.263 ± 0.509	Ref
1 nM	2.032 ± 0.514		4.628 ± 0.418 a	0.002
0.01 µM	1.973 ± 0.520		4.176 ± 0.598	0.101
0.1 µM	1.628 ± 0.842		3.994 ± 0.553	0.384
1 µM	1.960 ± 0.887		3.902 ± 0.669	0.429
10 µM	1.770 ± 0.783		2.995 ± 0.796	0.103

Abbreviations: Ref, reference; SD, standard deviation.

a *p* < 0.01 compared with the control group.

### Migration Assay: Boyden Chamber Assay

**Fig. 3:**
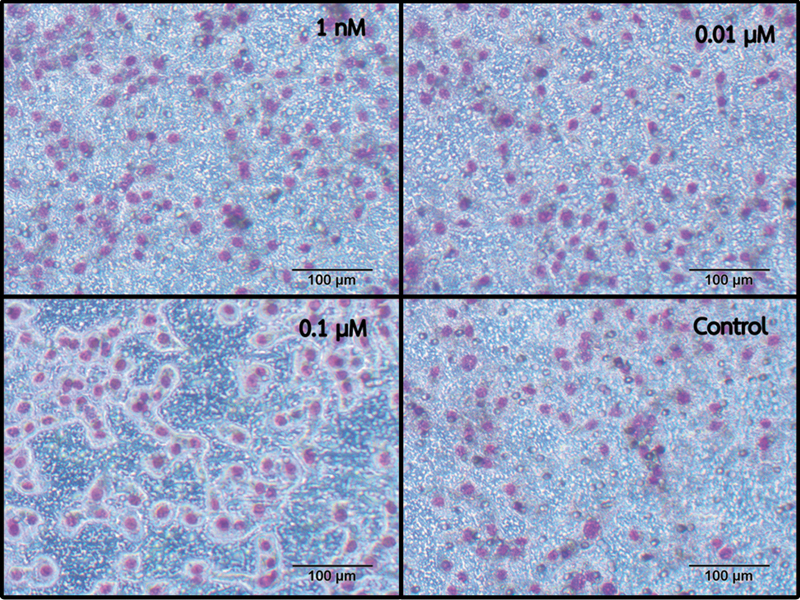
Micrographs show migrated cells on the bottom side of the transwell chambers, when cultured with 1 nM, 0.01 µM, 0.1 µM capsaicin, and without capsaicin (control), at 24 hours. The cells were stained with Giemsa and photographed at a 10× magnification.

In the Boyden chamber assay, we evaluated the migration or invasive capacity of RPC-C2A cells in response to various capsaicin solutions. The cells were seeded in the upper part of the membrane and treated with capsaicin concentrations of 1 nM, 0.01 µM, and 0.1 µM. After 24 hours, the cells that had migrated through the membrane and reached the other side were examined ( Fig. 3 ). The results of cell counting are presented in Table 2 . However, no significant difference in cell numbers was observed among all the treated groups when compared with the control group at the observation time ( *p* > 0.05).

**Table 2:** Counts of the migrated cells on the bottom side of the transwell chamber after 24 hours

	Number of migrated cells (mean ± SD)
**Control**	802 ± 138
**1 nM**	876 ± 355
**0.01 µM**	959 ± 198
**0.1 µM**	966 ± 439

Abbreviation: SD, standard deviation.

Note: No significant difference is observed among all groups.

### Determination of Alkaline Phosphatase Activity

In order to assess any effect on ALP expression induced by capsaicin, the activity of ALP enzyme was determined. Table 3 shows the ALP activity of RPC-C2A cells on day 5. The results indicate a significant increase in ALP activity, as measured by the absorbance value, in the 1 nM and 1 µM capsaicin-treated groups, with *p* < 0.001 and *p* = 0.021, respectively.

**Table 3:** Alkaline phosphatase activity of RCP-C2A cells on day 5 after treatment

On day 5	Absorbance value at 405 nm (mean ± SD)	*p-* Value
Control	0.227 ± 0.002	Ref
1 nM	0.250 ± 0.005 a	<0.001
0.01 µM	0.226 ± 0.018	1.000
0.1 µM	0.224 ± 0.017	1.000
1 µM	0.236 ± 0.006 ^b^	0.021

Abbreviations: Ref, reference; SD, standard deviation.

a *p* < 0.01, ^b^ *p* < 0.05, compared with the control group.

## Discussion

The present study aimed to investigate the effects of pure capsaicin extract on rat dental pulp cells, specifically focusing on cell viability, proliferation, migration, and ALP activity. The findings of this study provide valuable insights into the potential effect of capsaicin on dental pulp cell behavior and its potential application in dental pulp regeneration and treatment strategies.

RPC-C2A cells are clonal rat dental pulp cells characterized by high ALP activity. Due to its same characteristics as the isolated dental pulp, RCP-C2A cells have been widely used for cytotoxicity and ALP expression on dental material testing. 18 19 20 21 The selection of the established cell line was desirable because this cell line was easily manipulated and maintained in culture, the variability due to different donors was eliminated, and better reproducibility was achievable.

The cell viability and proliferation of capsaicin extract were evaluated using the MTT assay. The results showed that, at lower concentrations (less than 10 µM), capsaicin showed a stimulatory effect on cell proliferation at specific observation times. These results align with previous studies that have reported the proliferative effects of capsaicin on various cell types, including fibroblasts and osteoblasts. 22 23 24 It was reported that functional TRPV1, also known as a capsaicin receptor, could accelerate gingival epithelial cell proliferation. 24 Dental pulp cells were reported to express TRPV1. 15 25 It is most likely that the stimulatory effect on dental pulp cell proliferation may come from the activation of TRPV1. However, the mechanism of this phenomenon needs to be elucidated.

However, a higher concentration (100 µM) of capsaicin led to a significant reduction in cell viability, suggesting a cytotoxic effect. These findings are consistent with previous studies that have reported the cytotoxic or antiproliferative effects of high-concentration capsaicin on various cell types. 6 26 27 28 Therefore, it is important to consider the concentration and dosage of capsaicin when utilizing it in dental pulp therapies to ensure its safety and efficacy.

Furthermore, the wound healing assay and Boyden chamber assay were employed to evaluate the effect of capsaicin on cell migration. The wound healing assay revealed that pure capsaicin extract, particularly at a lower concentration (1 nM), promoted cell migration at 16 hours. The increased migration observed suggests that capsaicin has the ability to enhance the mobility and migration of dental pulp cells. These findings are consistent with previous studies demonstrating the migratory effects of capsaicin on fibroblasts, endothelial cells, and inflammatory cells. 29 30 31 The Boyden chamber assay further confirmed the invasive potential of dental pulp cells in response to capsaicin, as a higher number of cells migrated toward the capsaicin solution through the membrane at 24 hours, even without statistical significance. These results suggest that, despite no stimulatory effect on invasive capacity, capsaicin extract is likely to enhance cell migration in dental pulp cells, which could be advantageous in promoting the reparative processes within the dental pulp.

This study investigated the capsaicin's effects on ALP activity, which was reported to indicate the early stage of dentinogenesis and pulp repair. 32 33 34 The results demonstrated that capsaicin extracts at lower concentrations (1 nM and 0.01 µM) significantly increased ALP activity in dental pulp cells compared with the control group. This finding suggests that capsaicin has the potential to induce odontoblast-like differentiation and enhance dentin mineralization processes in dental pulp cells. Some researchers have reported the osteogenic and mineralization-inducing effects of capsaicin on osteoblasts and mesenchymal stem cells. 23 35 Our limited results on ALP activity alone might not be adequate to conclude the osteogenic differentiation induced by capsaicin. Hence, it is very interesting to investigate any other osteogenic markers further to prove this.

Apart from its enhanced effects on dental pulp, the analgesic, anti-inflammatory, and antibacterial effects of capsaicin can be advantageous, when used as a dressing medication onto the injured dental pulp. 36 37 38 As dental pulp biology has revealed a huge amount of information to understand possible pulp healing potential, vital pulp therapies have become more acceptable among dental professionals. 39 Therefore, vital pulp therapies with capsaicin medication could offer promising outcomes on dental pulp repair. Further studies are warranted to explore the underlying molecular mechanisms through which capsaicin affects dental pulp cells and to clarify its possibility in the osteogenic differentiation of dental pulp cells. 40

## Conclusion

Pure capsaicin extract with lower concentrations of less than 10 µM promoted cell proliferation, migration, and ALP activity. Our findings contribute to the understanding of capsaicin's effects on dental pulp cells and suggest its potential application in dental pulp repair.
